# The Non-Erythropoietic EPO Analogue Cibinetide Inhibits Osteoclastogenesis In Vitro and Increases Bone Mineral Density in Mice

**DOI:** 10.3390/ijms23010055

**Published:** 2021-12-21

**Authors:** Zamzam Awida, Almog Bachar, Hussam Saed, Anton Gorodov, Nathalie Ben-Califa, Maria Ibrahim, Albert Kolomansky, Jennifer Ana Iden, Liad Graniewitz Visacovsky, Tamar Liron, Sahar Hiram-Bab, Michael Brines, Yankel Gabet, Drorit Neumann

**Affiliations:** 1 Department of Cell and Developmental Biology, Sackler Faculty of Medicine, Tel Aviv University, Tel Aviv 6997801, Israel; Zamzam.awida@gmail.com (Z.A.); almogb19950@gmail.com (A.B.); hussamsaed07@gmail.com (H.S.); anton.1985@hotmail.com (A.G.); alloulnat@gmail.com (N.B.-C.); Maria_rulez1@hotmail.com (M.I.); alexkolomansky228@gmail.com (A.K.); liadv@mail.tau.ac.il (L.G.V.); 2 Department of Medicine A, Tel Aviv Sourasky Medical Center, Sackler Faculty of Medicine, Tel Aviv University, Tel Aviv 6423906, Israel; 3 Department of Anatomy and Anthropology, Sackler Faculty of Medicine, Tel Aviv University, Tel Aviv 6997801, Israel; jenniden@gmail.com (J.A.I.); tamarlrn@gmail.com (T.L.); saharurit@gmail.com (S.H.-B.); 4 Araim Pharmaceuticals, Tarrytown, NY 10591, USA; mbrines@araimpharma.com

**Keywords:** erythropoietin, cibinetide, osteoclasts, bone marrow derived macrophages (BMDM), EPOR, CD131

## Abstract

The two erythropoietin (EPO) receptor forms mediate different cellular responses to erythropoietin. While hematopoiesis is mediated via the homodimeric EPO receptor (EPOR), tissue protection is conferred via a heteromer composed of EPOR and CD131. In the skeletal system, EPO stimulates osteoclast precursors and induces bone loss. However, the underlying molecular mechanisms are still elusive. Here, we evaluated the role of the heteromeric complex in bone metabolism in vivo and in vitro by using Cibinetide (CIB), a non-erythropoietic EPO analogue that exclusively binds the heteromeric receptor. CIB is administered either alone or in combination with EPO. One month of CIB treatment significantly increased the cortical (~5.8%) and trabecular (~5.2%) bone mineral density in C57BL/6J WT female mice. Similarly, administration of CIB for five consecutive days to female mice that concurrently received EPO on days one and four, reduced the number of osteoclast progenitors, defined by flow cytometry as Lin^−^CD11b^−^Ly6C^hi^ CD115^+^, by 42.8% compared to treatment with EPO alone. In addition, CIB alone or in combination with EPO inhibited osteoclastogenesis in vitro. Our findings introduce CIB either as a stand-alone treatment, or in combination with EPO, as an appealing candidate for the treatment of the bone loss that accompanies EPO treatment.

## 1. Introduction

Erythropoietin (EPO) is a glycoprotein hormone that is produced mainly by the kidneys and plays a crucial role in regulating erythropoiesis [1]. The secreted hormone acts by binding to the EPO receptor (EPOR) on erythroid progenitor cells in the bone marrow (BM), and stimulates their proliferation, differentiation, and survival. [2]

Recombinant human EPO (rHuEPO) is administered clinically for the treatment of anemia that is secondary to chronic kidney disease, or for certain hematological malignancies [3,4,5,6].

In addition to hematopoietic cells, EPOR has also been detected in many other cell types and is associated with cytoprotective effects in these non-hematopoietic tissues [7,8,9,10,11,12]. While erythropoiesis is mediated by the canonical EPOR homodimer, the tissue protective effects of EPO are thought to act through the heteromeric innate repair receptor (IRR) [13,14], which is composed of EPOR and the β-common receptor subunit (βcR, CD131). This subunit is also used for signaling by other type 1 cytokine receptors, such as GM-CSF, IL-3, and IL-5 [15,16,17].

A number of non-hematopoietic EPO analogs have now been developed that selectively activate the IRR and confer tissue protection without a measurable effect on the EPOR-EPOR homodimer and, consequently, without eliciting erythropoiesis or the potential associated adverse effects [18]. One promising candidate is Cibinetide (pHBSP; ARA 290) which is an 11-amino acid peptide that is modeled from the three-dimensional structure of helix B of the EPO molecule [19]. Cibinetide interacts with the IRR and has demonstrated efficacy in a number of animal models of angiogenic potential, autoimmune diseases, neuropathy, and organ transplantation [20,21,22,23,24,25].

Bone is a highly dynamic tissue that undergoes continuous remodeling throughout life in a process involving the concerted actions of monocyte-derived osteoclasts that resorb mineralized tissue, and mesenchymal osteoblasts that deposit new bone [26,27,28].

The tight regulation of the osteoblastic and osteoclastic lineages is thus crucial for the control of bone turnover [29,30,31,32].

The discovery that EPO has pleiotropic roles in non-hematopoietic tissues has prompted an investigation of the role of EPO in bone homeostasis [33,34]. This is due to the proximity of the bone and hematopoietic cells in the bone marrow milieu as well as the documented crosstalk between the hematopoietic and skeletal systems [35,36]. In that respect, we, and others, have shown that high EPO levels (both exogenously administered as well as a result of over-expression) lead to a dramatic bone loss in mouse models [37,38,39,40,41,42]. 

In accordance with these results, a growing body of evidence now links high EPO levels to bone loss in humans [43,44].

Monocyte differentiation into osteoclasts is driven by the receptor activator for nuclear factor kappa B (RANK)/RANK ligand (RANKL)/osteoprotegerin (OPG) system, and macrophage colony stimulating factor (M-CSF, CD115) [45]. In the bone marrow, these preosteoclast macrophages express EPOR and recent studies from our lab indicate that EPO stimulates osteoclastogenesis by acting directly on osteoclast precursors [38]. However, the underlying molecular mechanism that is responsible for EPO-induced bone loss is still unknown.

This study was designed to investigate the role of the EPO-R forms in bone metabolism. For this purpose, we used the non-erythropoietic EPO analog Cibinetide, which acts exclusively on the EPOR/CD131 complex.

Here we present data suggesting that Cibinetide treatment in murine models is associated with a bone-preserving effect when injected as a single agent. Moreover, Cibinetide counteracts the stimulatory effect of EPO on preosteoclasts, making it a very promising anti resorptive agent, either when given alone or in combination with EPO.

## 2. Results

### 2.1. Cibinetide (CIB) Inhibits Osteoclastogenesis In Vitro, in a Dose Dependent Manner, without Cytotoxic Effects

**Figure 1:**
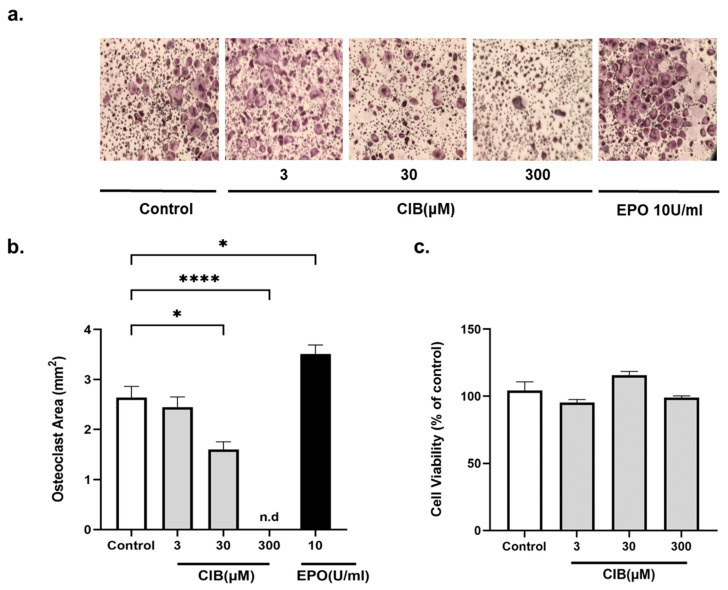
Cibinetide (CIB) attenuates osteoclast differentiation in vitro. BMDM were treated with Cibinetide in the presence of RANKL (50 ng/mL) and M-CSF (20 ng/mL). TRAP staining (images and TRAP^+^ area) of osteoclasts are shown at the end of differentiation (4–5 days). (**a**) Representative micrographs of TRAP^+^ osteoclasts. Original magnification at 2×. (**b**) Quantification of the total area of TRAP^+^ multinucleated cells. (**c**) BMDMs were cultured in the presence of different concentrations of Cibinetide (3, 30, 300 μM). After 48 h, MTT reagent was added to each well and the absorbance was measured at 560 nm. Data from at least three independent experiments are shown as mean ± SEM. * *p* < 0.05, **** *p* < 0.0001.

As the first step, we examined the effect of Cibinetide on the differentiation of bone marrow derived macrophages (BMDM) into osteoclasts as mediated by M-CSF and RANKL, which are both essential for osteoclast differentiation. TRAP staining was used to evaluate the area that was covered by osteoclasts, with treatment with EPO as the positive control for increased osteoclastogenesis [38]. Cibinetide suppressed osteoclastogenesis in a dose-dependent manner. At low Cibinetide levels (3 μM), osteoclastogenesis was similar to that which was observed in the control (M-CSF and RANKL only) cultures (Figure 1a,b). To exclude the possibility that inhibition of osteoclastogenesis by Cibinetide was due to the reduced viability of the osteoclast precursors, we examined the viability by MTT assay. The results confirmed that Cibinetide has no cytotoxic effects at concentrations that effectively inhibited osteoclast differentiation (Figure 1c). 

### 2.2. Cibinetide (CIB) Exerts Its Inhibitory Effects in the Early Stages of Osteoclastogenesis

**Figure 2:**
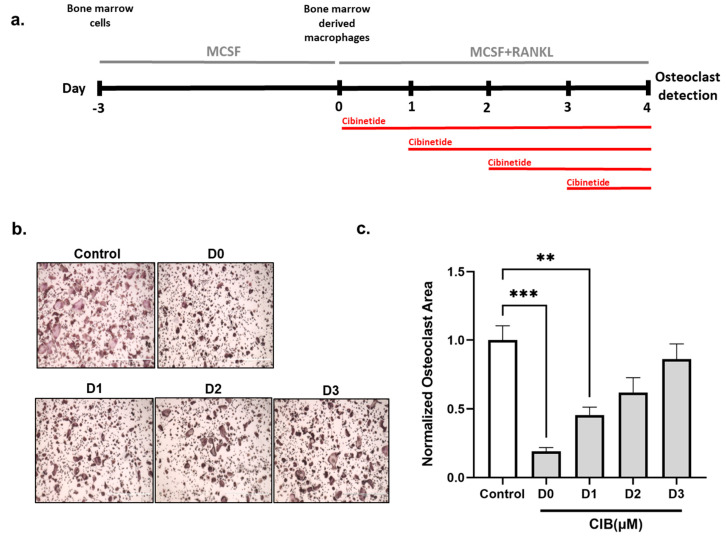
Cibinetide (CIB) inhibits osteoclastogenesis during the early stage of osteoclast differentiation in vitro. (**a**) Schematic representation of the experimental layout. (**b**) BMDM were treated with Cibinetide (150 μM) at four different time points (indicated by red lines) in the presence of RANKL (50 ng/mL) and M-CSF (20 ng/mL). After four days, TRAP staining was performed. (**c**) Quantification of the total area of TRAP^+^ multinucleated cells. Data from three independent experiments are shown as mean ± SEM. ** *p* < 0.01, *** *p* < 0.001.

To determine the stage at which Cibinetide inhibits osteoclastogenesis, BMDM that were previously cultured with M-CSF only were also supplemented with RANKL at Day 0, and were treated with Cibinetide (150 μM) at different times between days 0 and 4, as shown in the schematic representation (Figure 2a). Inhibition of osteoclastogenesis was most effective when Cibinetide was introduced into the cultures at day 0. Reduction of osteoclastogenesis by Cibinetide was far less pronounced when the compound was added at a later time point. These findings support the notion that the anti-osteoclastogenic effect of Cibinetide is manifested most strongly in the initial stages of osteoclast differentiation (Figure 2b,c).

### 2.3. Cibinetide (CIB) Suppresses the Expression of Osteoclast-Related Genes

**Figure 3:**
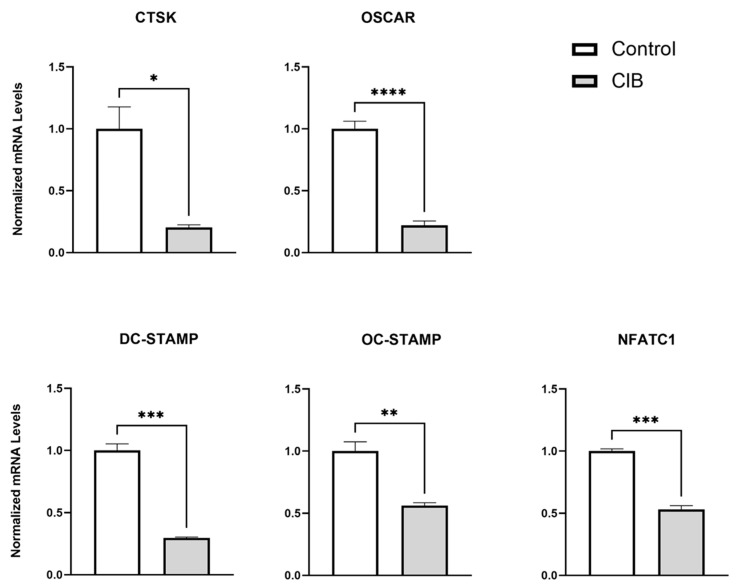
Cibinetide (CIB) downregulates osteoclast-specific genes’ expression. PCR analysis of osteoclast-specific gene expression in BMDMs stimulated with MCSF + RANKL for five days with or without Cibinetide (150 μM). The expression of Cathepsin K(CTSK), OSCAR, DC-STAMP, OC-STAMP, and NFATC1 were normalized to the housekeeping gene HPRT and then to the mean of the control group. The data are expressed as mean ± SEM of three independent experiments. * *p* < 0.05, ** *p* < 0.01, *** *p* < 0.001, and **** *p* < 0.0001.

To further examine the inhibitory effects of Cibinetide on osteoclastogenesis in response to RANKL, we examined the expression of the well-known osteoclast-related genes, Cathepsin K (CTSK), OSCAR, OC-STAMP, DC-STAMP, and NFATC1 [46]. The results (Figure 3) indicated that Cibinetide significantly inhibited the expression of all these osteoclast marker genes. These results are in accordance with the inhibitory effect of Cibinetide on osteoclastogenesis, as presented in Figure 1 and Figure 2. Collectively, these results confirm that Cibinetide suppresses osteoclastogenesis in vitro.

### 2.4. Cibinetide (CIB) Overrides the Pro-Osteoclastogenic Effects of LPS and EPO

**Figure 4:**
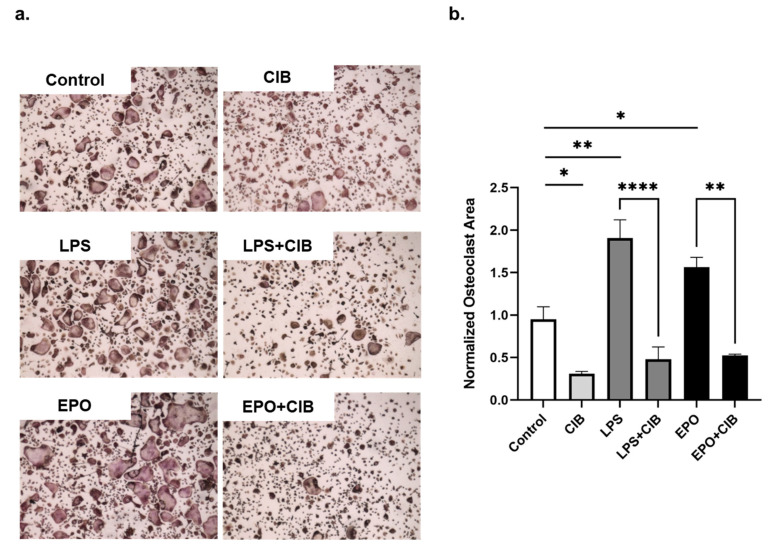
Cibinetide (CIB) counteracts the effect of EPO on preosteoclasts in vitro. BMDMs were treated with RANKL (50 ng/mL) along with EPO (10 U/mL), Cibinetide (150 μM), or EPO + Cibinetide. On the second day, LPS (10 ng/mL) was introduced to the RANKL or to the RANKL + Cibinetide cultures. On the fourth day, the multinucleated osteoclasts were stained for TRAP. (**a**) Representative micrographs of TRAP^+^ osteoclasts. Original magnification at 2×. (**b**) Quantification of the total area of TRAP^+^ multinucleated cells that were normalized to the values of the control group (RANKL). The data are mean ± SEM of three independent experiments. * *p* < 0.05, ** *p* <0.01, **** *p* < 0.0001.

To determine whether the anti osteoclastogenesis effect of Cibinetide can counter enhanced osteoclastogenesis, such as that which is conferred by EPO or LPS, Cibinetide was added to BMDM together with EPO or LPS as follows: BMDMs were treated with M-CSF and RANKL (50 ng/mL) together with EPO (10 U/mL) and/or Cibinetide (150 μM). On the second day, LPS (10 ng/mL) was introduced to the RANKL+M-CSF or to the RANKL+M-CSF+ Cibinetide cultures. On the fourth day, multinucleated osteoclasts were stained for TRAP. The results demonstrate that Cibinetide inhibits osteoclastogenesis even in the presence of LPS or EPO (Figure 4a,b).

### 2.5. Cibinetide (CIB) Increases Tissue Mineral Density (TMD) in Both Cortical and Trabecular Bone

**Figure 5:**
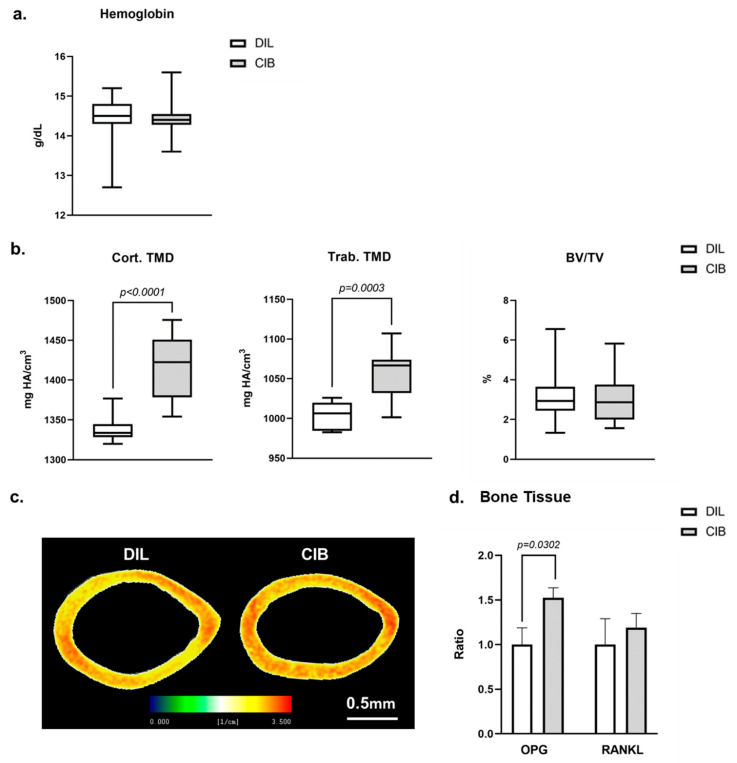
Cibinetide (CIB) increases tissue mineral density (TMD) in both cortical and trabecular bone. (**a**) Hemoglobin levels in Cibinetide versus diluent injected mice. (**b**) µCT analysis in the mid-diaphyseal cortical and distal trabecular femoral bone of diluent and Cibinetide injected 12-week-old female mice with respective representative images (**c**). Box plots represent median, quartiles and extremes. In (**c**), the color spectrum reflects the mineral density gradient across the section of the cortical bone. The upper limit of the scale (3.5/cm) corresponds to 1764 mg HA/cm^3^. (**d**) OPG and RANKL mRNA expression levels in whole bone from diluent vs. Cibinetide treated mice. In (**d**) ^ΔΔ^CT values are normalized to the diluent group; values are mean ± SEM. *N* = 9–10 mice in each group.

To investigate the effect of Cibinetide on bone density, we injected 12-week-old female C57BL/6J mice with Cibinetide (120 µg/kg × 3/week for 4 weeks). At the end of the fourth week, hemoglobin levels were measured and the femurs were analyzed by µCT. As expected, Cibinetide treatment did not have any effect on hemoglobin levels (Figure 5a). However, it did cause a 5.8% increase in the cortical TMD and a 5.2% increase in the trabecular TMD, while the trabecular bone fraction (BV/TV) was unchanged (Figure 5b,c). To gain more insight into the effect of Cibinetide on bone, we analyzed the expression level of OPG and RANKL in the whole bones of Cibinetide-treated and untreated mice. In line with the observed increase in TMD, Cibinetide treatment resulted in a significant 52.4% increase in the expression of OPG in the whole bone, with no change in the expression of RANKL (Figure 5d).

### 2.6. Cibinetide (CIB) Modulates Alkaline Phosphatase (ALP) and CD115 Expression on CD11b^−^ Bone Marrow Cells

**Figure 6:**
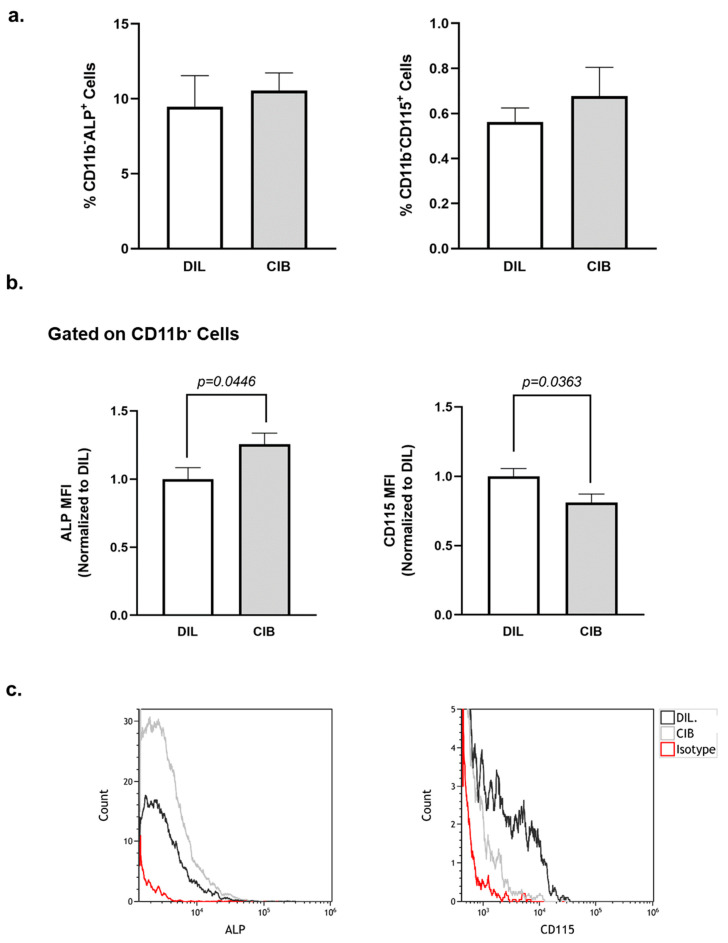
In vivo effects of Cibinetide (CIB) on bone cell precursors. Flow cytometry analysis of osteoblast (CD11b^−^ ALP^+^) and osteoclast precursors (CD11b^−^CD115^+^) of total bone marrow from diluent and Cibinetide injected mice. (**a**) Percentage of cells in the bone marrow. (**b**) Surface expression (mean fluorescence intensity (MFI)) of ALP and CD115 on CD11b^−^ cells in Cibinetide vs. diluent treated mice. (**c**) Representative overlay histograms of ALP and CD115 expression on CD11b^−^ cells. The data are mean ± SEM. *N* = 9–10 mice in each group.

As the next stage, we assessed the effect of Cibinetide on the percentage of preosteoblasts (CD11b^−^ALP^+^) as well as preosteoclasts (CD11b^−^CD115^+^) in the bone marrow [47]. The results (Figure 6a), indicate that Cibinetide treatment did not affect the percentage of these two cell populations in the bone marrow. Importantly, Cibinetide treatment resulted in a 25.59% increase in ALP expression and a 17.79% decrease in CD115 expression on the surface of the CD11b^−^ bone marrow cells (Figure 6b,c). These results further support a Cibinetide-mediated increase in osteoblast mineralizing activity and a decrease in the potential for osteoclast differentiation and are in accordance with our findings that are presented in Figure 5.

### 2.7. Cibinetide (CIB) Overrides the Pro-Osteoclastogenic Effects of EPO In Vivo

To evaluate the capacity of Cibinetide to counteract the effect of EPO on osteoclasts in vivo, we performed a short-term experiment in which 13-week-old female mice were administered Cibinetide (300 µg/kg) for 5 consecutive days, with 2 injections of 120 U EPO on days 1 and 4. These mice were compared to mice that were receiving either Cibinetide or EPO and also to untreated control mice. 

**Figure 7:**
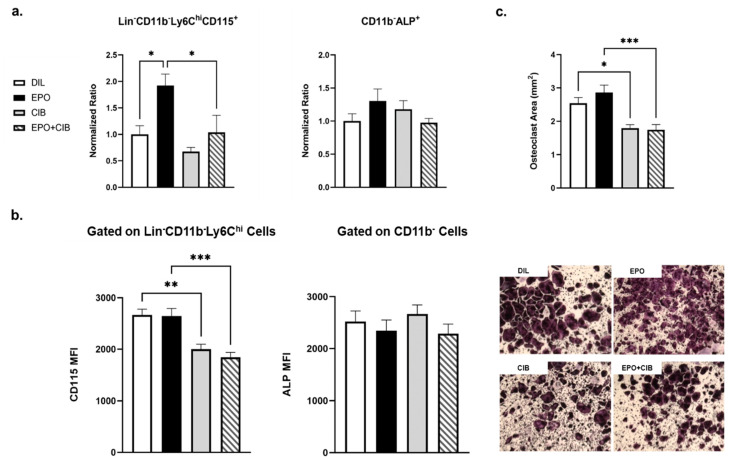
Reduced osteoclast progenitor numbers in combined EPO + Cibinetide (CIB) treatment. (**a**) Flow cytometry analysis of osteoclast progenitors-containing population Lin^−^ (CD3ε^−^, B220^−^, Ly6G^−^, and TER119^−^)CD11b^−^ Ly6C^hi^CD115^+^ cells, and osteoblast precursors CD11b^−^ALP^+^. The data are mean ± SEM of values that are normalized to the diluent group. (**b**) Surface expression (mean fluorescence intensity (MFI)) of CD115 and ALP in diluent vs. EPO, Cibinetide or EPO + Cibinetide-treated mice. (**c**) Total area of multinucleated TRAP^+^ osteoclasts grown ex vivo with M-CSF and RANKL from non-adherent bone marrow cells that were isolated from mice treated with either diluent, EPO, Cibinetide, or EPO + Cibinetide. Right, representative images acquired at ×2 magnification. All data are mean ± SEM. *N* = 7 in each group. * *p* < 0.05, ** *p* < 0.01, *** *p* < 0.001.

Flow cytometry analysis revealed an 81.06% increase in the number of osteoclast progenitors, defined as Lin^−^CD11b^−^Ly6C^hi^CD115^+^ [48,49,50,51], in the EPO injected mice. However, this was reduced by 42.8% in the EPO + Cibinetide-injected mice compared to the EPO-injected mice, without any change in the numbers of osteoblast precursors (Figure 7a). Moreover, Cibinetide treatment downregulated CD115 expression on the osteoclast precursors both when given alone and in combination with EPO, while EPO had no effect (Figure 7b). As the next step, we performed an ex vivo osteoclastogenesis assay where a fixed number of BM cells that were collected from mice in each treatment group were cultured in the presence of M-CSF + RANKL. The number of osteoclasts that were measured in this assay reflects the proportion of osteoclast progenitors in the mice at the end of the treatment. The results indicated that cultures that were derived from the EPO-treated mice exhibited a higher degree of osteoclastogenesis than the control cultures, although the difference did not reach statistical significance. Of note, a single injection of EPO at a higher dose (180 U) resulted in an increase both in the proportion of osteoclast progenitors and in the level of CD115 surface expression, as measured after 48 h (Supplementary Materials, Figure S1)). Importantly, the level of osteoclastogenesis in cells from the Cibinetide and EPO + Cibinetide-injected mice was significantly lower than that from either the control or EPO groups (Figure 7c). 

With this short-term treatment, no changes in the bone parameters were detected by µCT in any of the treatment groups (Supplementary Materials, Figure S2).

### 2.8. EPO Maintains Erythropoietic Activity in the Presence of Cibinetide (CIB)

**Figure 8:**
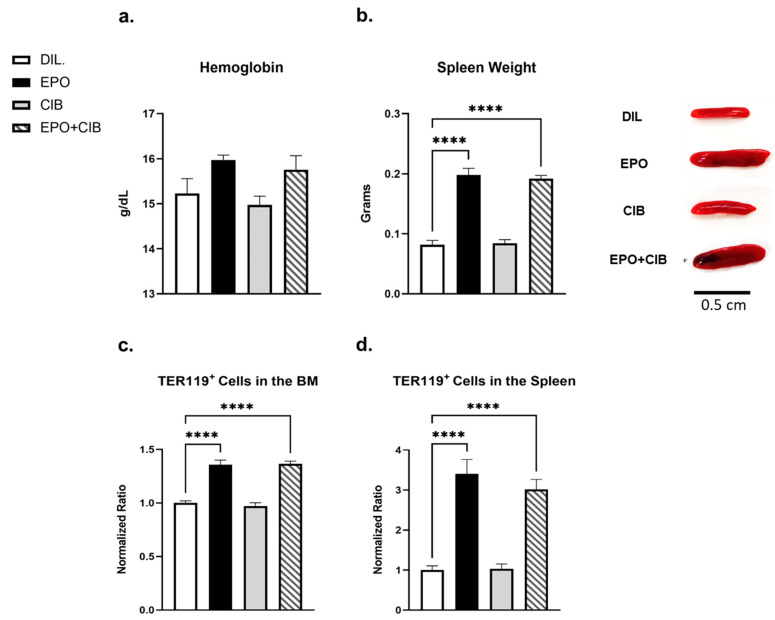
Cibinetide (CIB) does not interfere with the erythropoietic response to Erythropoietin. (**a**) Hemoglobin levels. (**b**) Average weights of the spleens with respective representative photographs from untreated, EPO, Cibinetide, EPO + Cibinetide-treated mice. (**c**,**d**) Flow cytometry analysis of TER119^+^ erythroid progenitors in the bone marrow (**c**) and in the spleen (**d**). Values on the Y axis represent the normalized ratio of the Ter119^+^ cells in each treatment group relative to the diluent control. Values are mean ± SEM. *N* = 7 mice in each group. **** *p* < 0.0001.

To exclude the possibility that Cibinetide antagonizes the erythropoietic effects of EPO on erythroid cells, we measured hemoglobin levels and spleen weights. In addition, we evaluated the levels of TER119^+^ erythroid progenitor cells in the spleen and bone marrow by flow cytometry. As expected, the EPO treatment resulted in a significant increase in all these parameters and none of the erythropoietic changes that were mediated by EPO were affected by Cibinetide (Figure 8).

## 3. Discussion

While the beneficial anti-inflammatory effects of Cibinetide, a selective IRR agonist, have been well-documented in multiple systems for over a decade now [52], there is no record of the effect on the skeletal system, which is one of the most studied targets of EPO.

This study is the first to address the in vitro and in vivo effects of Cibinetide on bone metabolism, either alone or in combination with EPO.

Our results reveal that Cibinetide can counteract the stimulatory effects of EPO on the monocytic-lineage by decreasing the pool of osteoclast precursors that were defined as Lin^−^ CD11b^−^Ly6C^hi^CD115^+^ in the bone marrow, and by further decreasing the expression of CD115 in this population. The observation that this is achieved without interference with the erythropoietic activity of EPO, reflects the specificity of the effect on osteoclast progenitors in the bone marrow.

The finding that Cibinetide acts in the early stages of osteoclast differentiation is evidenced by the downregulation of the key transcription factor NFATC1, as well as suppression of the osteoclast differentiation-related genes CTSK, OSCAR, DC-STAMP, and OC-STAMP. Interestingly, EPO has previously been reported by us [38] and others [53,54] to have an osteoclast stimulatory effect.

These seemingly opposing effects of the two EPOR ligands on preosteoclasts in vitro motivated us to examine the interplay between the two ligands during osteoclastogenesis. Importantly, the results revealed that the inhibitory effect of Cibinetide could negate the stimulatory effect that was induced by EPO.

Considering the potent anti-inflammatory activities of Cibinetide in LPS-activated macrophages [24,55,56], and the fact that LPS is a potent inducer of inflammation and inflammatory bone loss [57], our finding that Cibinetide mitigates LPS-induced osteoclastogenesis may have significant clinical applications, for example in inflammation-induced osteolysis (e.g., [58,59,60]). It should be noted that the components of the heteromeric EPOR and CD131 complex are usually intracellular in the non-inflammatory state and typically are not expressed by healthy tissues although they are rapidly induced under stress conditions [61]. The robust in vitro anti-osteoclastogenic effects of Cibinetide (in the presence of RANKL and M-CSF), prompted us to investigate whether Cibinetide can also confer a bone preserving effect in vivo, in healthy mice.

The administration of Cibinetide to wild-type female mice resulted in a significant increase in TMD, which is an important contributor to bone mineral density (BMD) in cortical and trabecular bone, while the BV/TV remained unchanged after Cibinetide treatment. TMD is a measure of the mineral content inside the trabecular and cortical bone whereas BV/TV measures the amount of trabecular and cortical bone over the total volume of the region of interest. At the resolution that was employed here (10 µm), TMD will thus reflect the mineral density as well as the microscopic porosity (pores of <10–15 µm) as the measured bone volume will include these small voids that cannot be detected due to the resolution. This microscopic porosity includes osteocyte lacunae [62]. In our study, the Cibinetide-induced increase in TMD may result from increased mineralization of the bone tissue and/or narrowing of the osteocyte lacunae. BMD combines both TMD and BV/TV. It should be noted that despite the seemingly low increase in TMD (~5%), this is a clinically relevant finding since a 5–10% decline is associated with approximately 50–100% higher fracture rates [63].

Interestingly, the Cibinetide results are very different from the effect that was observed after exogenous EPO administration in mice, where a massive decrease in trabecular bone fraction is evident albeit with no effect on TMD (data not shown).

The increased expression levels of OPG in the bone tissue of Cibinetide treated mice, are a possible mechanism for the reduced osteoclastogenesis, reduced bone resorption and increased TMD.

This assumption is further supported by a recent study reporting that EPOR regulates OPG expression in osteo-progenitors and regulates osteoblast function and osteoblast-mediated osteoclastogenesis via the RANKL/OPG axis [42]. The question of whether EPO acts on osteoblasts via the EPOR/CD131 heterodimer remains to be addressed. 

Despite the similar proportions of preosteoblasts and preosteoclasts in the bone marrow of Cibinetide-injected mice, we found an increased surface expression of alkaline phosphatase, an early osteogenic marker of bone formation and mineralization on preosteoblasts. In addition, there was a decrease in the expression of CD115, one of the receptors for osteoclastogenic factors which acts as a potent stimulator of RANK expression on preosteoclasts [64]. In line with these findings, the downregulation, blockade, or depletion of CD115 has been shown to suppress the formation and activity of osteoclasts and to attenuate the pathological bone resorption that is seen in inflammatory bone destruction and osteoporosis [65,66,67,68]. Similarly, TLR ligands mediate the downregulation of CD115 from the cell surface and suppress osteoclastogenesis [69].

Taken together, our data provide evidence that Cibinetide acts on preosteoblasts to increase bone formation and also reduces bone resorption capacity by suppressing the differentiation of preosteoclasts to osteoclasts.

Another cytokine receptor that shares the β chain (CD131) of the heteromeric complex is IL-3. In one study, IL-3 was shown to decrease the expression of CD115 glycoprotein and mRNA in a murine myeloid precursor cell line [70] and in another study, it was found to inhibit human osteoclastogenesis and bone resorption through downregulation of CD115 and diverting the cells to the dendritic cell lineage [71]. These findings raise certain questions regarding the role of the CD131 subunit in bone remodeling.

In this context, it is interesting that CD131 knockout mice exhibit normal development, although they develop pulmonary peribronchovascular lymphoid infiltrates and areas resembling alveolar proteinosis [72], and the numbers of eosinophils are reduced in the peripheral blood and bone marrow. Notably, the tissue-protective properties of EPO do not function in these animals although they display normal hematopoiesis [13,55]. In another study, the very challenging conditional deletion of CD131 in a specific subpopulation through the myeloid lineage CCR2^+^Ly6C^hi^ was achieved and used to study experimental autoimmune encephalomyelitis (EAE) [73].

In the future, we predict that a similar approach of generating osteoclast-specific and osteoblast-specific conditional CD131 knockout may strengthen our conclusion that the heteromer EPOR/CD131 mediates the effects of EPO and Cibinetide in these bone cells.

The current study used only a short-term treatment of the combination of Cibinetide and EPO. Our choice of a five-day treatment regimen was based on our findings of a very early and rapid increase in osteoclast progenitors two days after a single EPO injection (2.25-fold increase vs. untreated). With our short duration of treatment, we could not detect any changes in bone microarchitecture in any of the treated groups, but we did observe significant differences in the osteoclast lineage, in line with the skeletal effects of CIB that were observed (Figure 5).

The findings that are reported here add more complexity to the notion that the two ligands induce similar tissue protective activities and differ only in their erythropoietic capacity. To our knowledge, this is the first scenario in which Cibinetide and EPO have opposing effects. Whether this is due to different pharmacokinetics or to diverging signaling pathways remains to be elucidated. 

In this context, it should be noted that although Cibinetide has a short plasma half-life (~2 min) [74], it has sustained biological effects [52], even when injected with EPO, which is a molecule with a long half-life (4–6 h) [52]. 

In light of the fact that all of the currently used anti-resorptive drugs for the treatment of osteoporosis have adverse side effects, an intense focus has been directed toward developing new therapeutic agents that can inhibit bone resorption and enhance bone formation [75]. 

Our data introduce Cibinetide as an appealing candidate for the treatment of bone loss either as a stand-alone therapy for osteolytic pathologies ranging from cancer to rheumatoid arthritis and osteoporosis, or in combination with EPO for patients with anemia or cancer. The results that are presented here suggest that treatment with Cibinetide and EPO may prevent or attenuate bone loss while preserving the erythropoietic actions of EPO.

## 4. Materials and Methods

### 4.1. Materials

Alpha-MEM and fetal bovine serum (FBS), were purchased from Rhenium (Modiin, Israel) and culture plates were from Corning (New York, NY, USA). LPS from the *E. coli* strain 0127:B8 (Sigma) was reconstituted in sterile double distilled water (DDW) as a 1 mg/mL stock solution and kept at −20 °C. Dulbecco’s Phosphate Buffered Saline (DPBS) was purchased from Biological Industries. As a source of M-CSF, we used supernatant from CMG 14-12 cells containing 1.3 μg/mL M-CSF [38,76]. RANKL was purchased from R&D Systems, Minneapolis, MN, USA. The peptide Cibinetide was kindly provided by Araim Pharmaceuticals, Inc. (Ossining, NY, USA). Erythropoietin (EPO) was obtained from GMP-manufactured sterile syringes containing rHuEPO (Epoetin alfa, Eprex^®^) as used for patient care. These were kindly provided by Janssen Cilag, Israel, and were employed throughout this study.

### 4.2. Animals

Female wild-type mice of the inbred strain C57BL/6J-RccHsd, aged 8–13 weeks, were purchased from Envigo (Jerusalem, Israel) and housed at the Tel-Aviv University animal facility. Animal care and all procedures were in accordance with, and with the approval of, the Tel Aviv University Institutional Animal Care and Use Committee (Permit number 01-19-032). 

### 4.3. Cell Culture

In vitro osteoclastogenesis: Bone marrow cells were harvested from the femurs and tibias of 8- to 13-week-old female mice. The cells were seeded on tissue-culture treated plates in standard medium (α-MEM supplemented with 10% fetal bovine serum). On the following day, the non-adherent cells were seeded in non–tissue culture-treated plates in standard medium supplemented with 100 ng/mL M-CSF [77], which induces cell proliferation and differentiation into preosteoclasts.

For the osteoclastogenesis assay, preosteoclasts were plated in 96-well plates (8000 cells per well) with standard medium that was supplemented with 20 ng/mL M-CSF and 50 ng/mL RANKL (R&D Systems), which was replaced every 2 days. Treatments in culture (EPO, Cibinetide, LPS) were added together with RANKL, as indicated. On the 4–5th day, the multinucleated osteoclasts [78] were stained using a tartrate-resistant acid phosphatase (TRAP) [79] kit (Sigma-Aldrich, St Louis, MO, USA) and the relative TRAP-positive surface was measured using ImageJ software.

Ex vivo osteoclastogenesis: The bone marrow cells were harvested from the femurs and tibias of 13-week-old female mice and were then seeded into tissue culture-treated plates in standard medium and allowed to attach overnight. Non-adherent cells were plated into 96 well plates in standard medium that was supplemented with M-CSF (in the form of 2% *v/v* culture supernatant from CMG 14–12 cells), and 50 ng/mL recombinant murine RANKL. The medium was replaced every other day. After 6–7 days, the cells were stained for tartrate-resistant acid phosphatase (TRAP) and the relative TRAP-positive surface was measured using ImageJ software.

### 4.4. Microcomputed Tomography (microCT)

The femurs (one per mouse) were examined using the µCT50 system (Scanco Medical AG, Wangen-Brüttisellen, Switzerland) [80,81]. Briefly, scans were performed at a 10 µm resolution, 90 kV energy, 114 mA intensity, and 1100 msec integration time. The mineralized tissues were segmented by a global thresholding procedure following Gaussian filtration of the stacked tomographic images [82]. Trabecular bone parameters were measured in the secondary spongiosa of the distal femoral metaphysis. Cortical parameters were determined in a 1 mm height ring in the mid-diaphyseal region.

### 4.5. Hemoglobin Levels

Hemoglobin (Hgb) levels were measured in venous blood (that was drawn from the facial vein) by means of a “Mission Plus” hemoglobin/hematocrit meter (Acon, San Diego, CA, USA).

### 4.6. MTT (3-(4,5-Dimethylthiazol-2-yl)-2,5-Diphenyltetrazolium Bromide) Assay

Cell viability was determined using an MTT assay, essentially as described previously [83]. The bone marrow-derived macrophages (BMDM) were seeded into 96-well plates (1× 10^4^ cells/well) and then incubated with 100 ng/mL M-CSF in the presence of various concentrations of Cibinetide. After 48 h of incubation, MTT (0.5 mg/mL) was added to each well for 3 h. At the end of the incubation, the insoluble formazan products were dissolved in acidic isopropanol, and absorbance at 560 nm was measured to assess the number of viable cells (proliferation and cytotoxicity).

### 4.7. Flow Cytometry

The bone marrow (BM) cells were flushed from the femurs or tibias, and the red blood cells were lysed using ACK lysis buffer (Quality Biological, Gaithersburg, MD). The cells were then stained for 20 min at 4 °C with conjugated anti-mouse antibodies (see Table 1 for a list of the antibodies that were used). After this time, cells were washed with PBS containing 1% FBS and analyzed by either Gallios or Cytoflex flow cytometers and Kaluza or CytExpert software (all from Beckman Coulter, Indianapolis, Indiana 46268, USA).

### 4.8. Real-Time RT PCR

Total RNA was extracted from BMDM using the TriRNA Pure kit (Cat#TRPD200, Geneaid, New Taipei City, Taiwan) and cDNA was synthesized using the qScript cDNA synthesis kit (Quantabio, MA, USA). “Real-time” quantitative PCR (RQ-PCR) was performed on a StepOnePlus instrument using the SYBR Green reagent (both from Applied Biosystems, CA, USA). The relative gene expression was calculated using the ΔΔCT method following normalization to the expression of HPRT as a housekeeping gene. 

The primers that were used for PCR were as follows: (F, forward; R, reverse): 

**DC-STAMP**, F TCCTCCATGAACAAACAGTTCCAA; **DC-STAMP**, R AGACGTGGTTTAGGAATGCAGCTC; **OC-STAMP**, F TTGCTCCTGTCCTACAGTGC; **OC-STAMP**, R GCCCTCAGTAACACAGCTCA; **NFATC1**, F GAGTACACCTTCCAGCACCTT; **NFATC1**, R TATGATGTCGGGAAAGAGA; **HPRT**, F TCCTCCTCAGACCGCTTTT; **HPRT**, R CCTGGTTCATCATCGCTAATC; **OSCAR**, F CGTGCTGACTTCACACCAAC; **OSCAR**, R GGTCACGTTGATCCCAGGAG; **Cathepsin K**, F GATACTGGACACCCACTGGGA; **Cathepsin K**, R CATTCTCAGACACAATCCAC; **RANKL**, F CATTCTCAGACACAATCCAC; **RANKL**, R ACATCCAACCATGAGCCTTC; **OPG**, F GAGACACAGCTCACAAGAGCAA; **OPG**, R GCTTTCACAGAGGTCAATGTCTT.

### 4.9. Statistical Analysis

The values are expressed as mean ± SEM (standard error of the mean). A Student’s *t*-test was used for calculating the statistical significance when comparing two groups of variables. In experiments with >2 groups of variables, a one-way ANOVA was applied. The level of statistical significance was set at *p* ≤ 0.05. Asterisks between bars indicate significant differences between two groups (* *p* ≤ 0.05, ** *p* ≤ 0.01, *** *p* ≤ 0.001, and **** *p* ≤ 0.0001). All statistical analyses were performed using Prism 9 (GraphPad). 

## Figures and Tables

**Table 1:** Antibodies used for flow cytometry analysis.

Antibody	Source	Identifier
TER-119-APC	BioLegend	Cat#: 116211
CD71-PE	BioLegend	Cat#: 113807
CD11b-APC	BioLegend	Cat#:101211
CD115-PE	Miltenyi Biotec	Cat#:130112828
LY6G-FITC	BioLegend	Cat#: 127605
TER119-FITC	BioLegend	Cat#: 116205
CD3ε-FITC	BioLegend	Cat#: 100305
B220-FITC	BioLegend	Cat#:103205
LY6C- PerCP/Cy5.5	BioLegend	Cat#: 128011
Alkaline Phosphatase (ALPL)	R&D systems	Cat#: AF2910
CD115-APC	eBioscience	Cat#: 14115282
Goat IgG (H+L)-PE	R&D systems	Cat#: F0107

## Supplementary Materials

The following are available online at www.mdpi.com/article/10.3390/ijms23010055/s1.
