# Supplementary figures and images for: The Non-Erythropoietic EPO Analogue Cibinetide Inhibits Osteoclastogenesis In Vitro and Increases Bone Mineral Density in Mice
Source: Int J Mol Sci. 2021 Dec 21;23(1):55. doi: 10.3390/ijms23010055 (PMC8744753; doi:10.3390/ijms23010055)

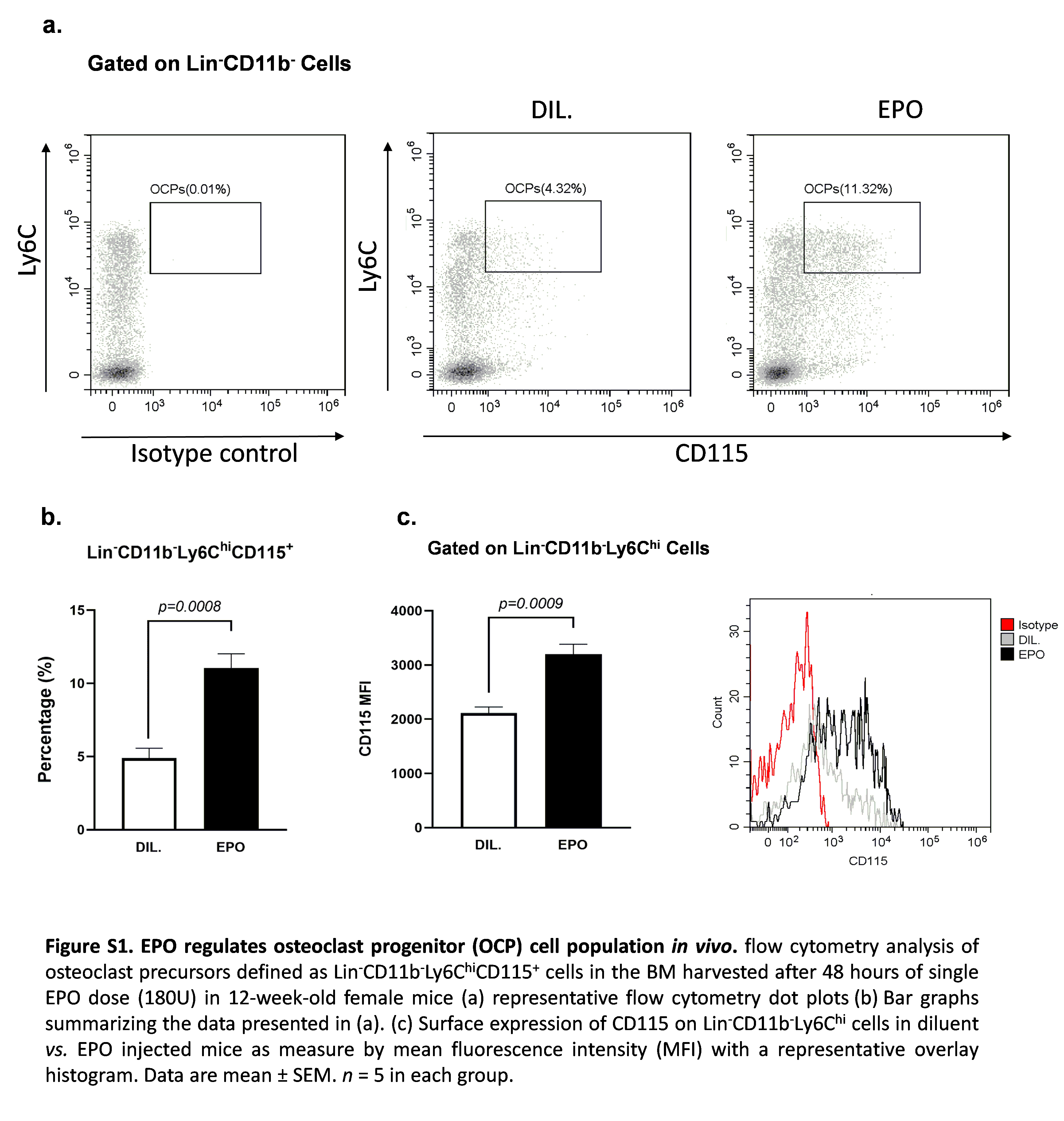

Supplement: Supplementary file 1 [file ijms-23-00055-s001.zip › ijms-1497919-supplementary/S1.bmp]

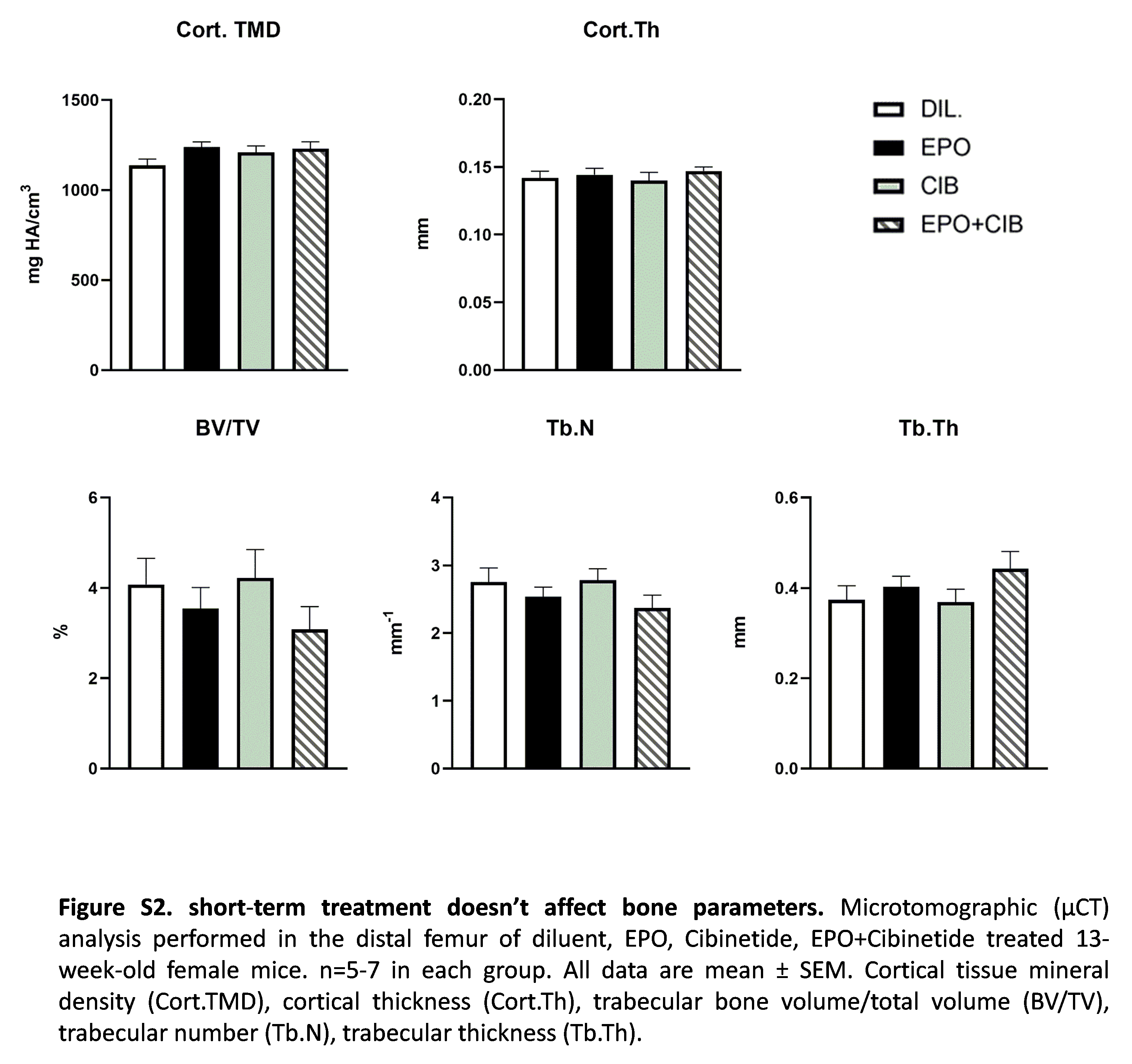

Supplement: Supplementary file 1 [file ijms-23-00055-s001.zip › ijms-1497919-supplementary/S2.bmp]
